# Development of an Efficient and Generalized MTSCAM Model to Predict Liquid Chromatography Retention Times of Organic Compounds

**DOI:** 10.34133/research.0607

**Published:** 2025-02-07

**Authors:** Mengdie Fan, Chenhui Sang, Hua Li, Yue Wei, Bin Zhang, Yang Xing, Jing Zhang, Jie Yin, Wei An, Bing Shao

**Affiliations:** ^1^ National Key Laboratory of Veterinary Public Health Security, College of Veterinary Medicine, China Agricultural University, Beijing Key Laboratory of Detection Technology for Animal-Derived Food Safety, and Beijing Laboratory for Food Quality and Safety, Beijing 100193, China.; ^2^ Beijing Key Laboratory of Diagnostic and Traceability Technologies for Food Poisoning, Beijing Center for Disease Prevention and Control, Beijing 100013, China.; ^3^ School of Public Health, Capital Medical University, Beijing 100069, China.; ^4^ National Engineering Research Center of Industrial Wastewater Detoxication and Resource Recovery, Research Center for Eco-Environmental Sciences, Chinese Academy of Sciences, Beijing 100085, China.

## Abstract

Accurate prediction of liquid chromatographic retention times is becoming increasingly important in nontargeted screening applications. Traditional retention time approaches heavily rely on the use of standard compounds, which is limited by the speed of synthesis and manufacture of standard products, and is time-consuming and labor-intensive. Recently, machine learning and artificial intelligence algorithms have been applied to retention time prediction, which show unparalleled advantages over traditional experimental methods. However, existing retention time prediction methods usually suffer from the scarcity of comprehensive training datasets, sparsity of valid data, and lack of classification in datasets, resulting in poor generalization capability and accuracy. In this study, a dataset for 10,905 compounds was constructed including their retention times. Next, an innovative classification system was implemented, classifying 10,905 compounds into a 3-tier hierarchy across 141 classes, based on functional group weighting. Then, data augmentation was performed within each category using simplified molecular input line entry system (SMILES) enumeration combined with structural similarity expansion. Finally, by training the optimal quantitative structure–retention relationship (QSRR) models for each category of compounds and selecting the best-fitting model for prediction via discriminant analysis during the prediction period, a novel and universal high-throughput retention time prediction model was established. The results demonstrate that this model achieves an *R*^2^ of 0.98 and an average prediction error of 23 s, outperforming currently published models. This study provides a scientific basis for high throughput and rapid prediction of unknown pollutants, data mining, nontargeted screening, etc.

## Introduction

With the rapid development of technology and industry, 279 million chemical substances have been cataloged in the Chemical Substance Database (CAS REGISTRYSM) as of June 2024 [1], with an annual increase rate of more than 10% for chemicals since 2009 [2]. However, only 3% of the chemicals in the market have comprehensive evaluation data, meaning that the characteristics of more than 90% of these compounds remain unclear, posing an unprecedented challenge for chemical identification, analysis, and risk control [3,4].

Retention time (RT) prediction is an important physicochemical parameter used for identification of compounds [5], which employs machine learning (ML) and artificial intelligence algorithms such as graph neural networks (GNNs) [6–9], artificial neural networks (ANNs) [10–12], random forest [5,13], support vector regression (SVR) [14–16], XGBoost [17–20], and their combination [21–24], to achieve automated, high-throughput, rapid prediction of compound RTs. However, in the process of building RT prediction models, a comprehensive and diverse training dataset, which is essential for model accuracy, is lacking. To date, the SMRT dataset has been the most commonly used dataset for the construction of compound RT prediction models [7,13], but it has certain limitations, such as a lack of generalization across different compound types [17,25] and a lack of interoperability between different laboratory operating conditions [7]. Therefore, it is essential to construct a comprehensive and generic training dataset.

RTs vary greatly for compounds with different structures [5,26]. However, current models have usually overlooked the impact of structural diversity on chromatographic retention behavior, increasing the interference arising from irrelevant variables and resulting in the reduction of predictive accuracy [10], which highlights the importance of introducing compound classification in the construction of RT prediction models [27,28]. However, the introduction of compound classification can lead to data sparsity, which can diminish the model’s generalization capability [29]. Therefore, it is vital in the implementation of compound classification strategies that they be complemented by data augmentation techniques [30]. In recent years, researchers have applied simplified molecular input line entry system SMILES enumeration (SE) or molecular similarity augmentation to expand the dataset’s volume [18–21], which provides a clue to the handling of data sparsity.

To tackle the challenges in chemical analysis, including compound diversity, the time-consuming and labor-intensive nature of traditional experimental methods, and standard compound acquisition difficulties, a universal high-throughput RT prediction model was developed in this study. This work has the following features: (a) a proprietary database has been constructed, comprising 10,905 compounds annotated with RTs under specific chromatographic conditions; (b) 10,905 compounds were classified into 3 levels and 141 classes based on their functional group weighting; and (c) the training dataset was augmented by employing SE and molecular similarity augmentation. This study will provide a powerful analytical tool for the rapid identification of unknown pollutants, structure elucidation and pollutant supervision, data mining, and nontargeted screening.

## Results

### Overall pipeline

**Fig. 1.**
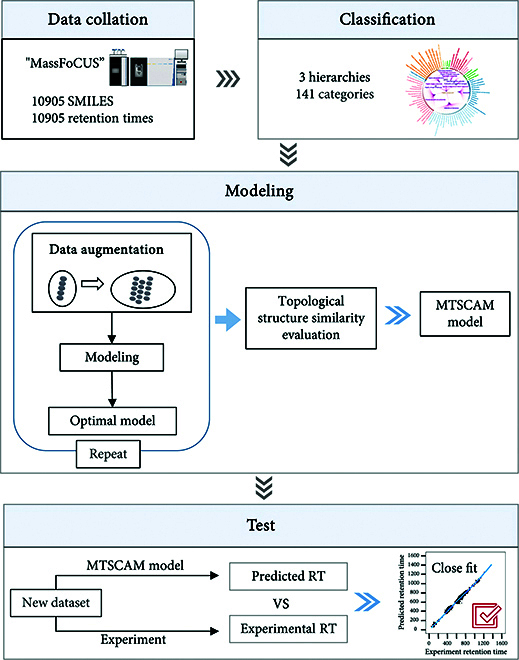
Model overview diagram.

An overview of the MTSCAM model is shown in Fig. 1, which contains 4 parts: data collation, classification, modeling, and testing. First, a database named MassFoCUS was constructed, including 10,905 small-molecule compounds. The database includes SMILES strings for each compound, and their RTs are measured under uniform experimental conditions. The MTSCAM model employs the Rdkit package to translate the SMILES strings of the datasets into SMARTS, thus enabling topology-based chemical classification within the MassFoCUS dataset via the ClassyFire platform [27].

**Fig. 2.**
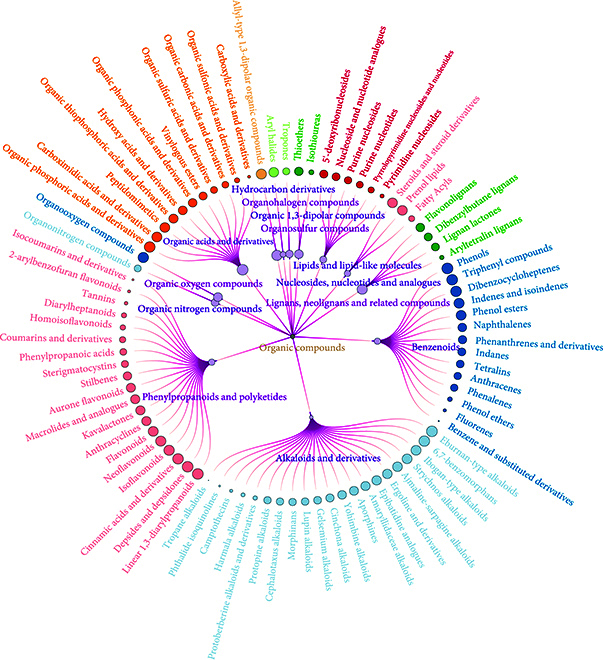
Classification of chemical substances in the MassFoCUS dataset.

Second, the 10,905 organic compounds were classified into 141 subclasses under 13 superclasses, based on the functional group categories and their corresponding weight coefficients in ClassyFire (classification information is shown in Fig. 2). RT prediction models were developed for each subclass-specific dataset. During the pretraining period, datasets for each subclass were randomly partitioned into training, validation, and test sets at an 8:1:1 ratio.

Third, a data augmentation strategy that combines SE with active learning based on molecular topology similarity augmentation, named topology-guided active learning (Topo-AL), was employed to enrich the training set. This approach traversed the SMILES strings of the training subset, enumerated additional SMILES expressions, and identified compounds across the full dataset with a structural similarity threshold exceeding 0.5. Initially, compounds identical to those in the test set were excluded. Following this, feature descriptors were derived from the SMILES expressions of the compounds, and feature selection and extraction processes were conducted. The models underwent pretraining with a suite of 7 ML algorithms, followed by evaluation on the test dataset. The optimal prediction submodel of retention time (OPSRT) model was identified for its superior prediction accuracy. Building upon this, a molecular topology prediction network was constructed by integrating a structural property discriminant analysis (SPDA) model with the pretrained OPSRT model. Fourth, validation with novel compounds revealed that the MTSCAM model’s predicted RTs were more closely aligned with those obtained from actual laboratory experiments than those derived by existing state-of-the-art models.

### Database construction and substance classification

RTs for 10,905 food chemical hazards were obtained via high-performance liquid chromatography–high-resolution mass spectrometry (HPLC-HRMS). The acquisition methodology is detailed in Materials and Methods. Pure standards of the compounds were obtained and combined to build the database. This database includes a wide variety of toxic substances, such as pesticides, veterinary drugs, additives, biotoxins, pharmaceuticals, psychoactive substances, and other environmental contaminants. These molecules are accessible on the MassFoCUS platform (http://scms.cau.edu.cn/).

Then, the 10,905 chemically hazardous substances were classified based on their functional groups and molecular weights, resulting in 13 secondary classifications and 141 tertiary classifications. The number of compounds within each class varies from tens to thousands, and these are utilized to train individual OPSRT models. The classification outcomes are depicted in Fig. 2, which delineates the 13 major secondary classes: organoheterocyclic compounds, benzenoids, organic nitrogen compounds, organic acids and derivatives, organic oxygen compounds, organosulfur compounds, phenylpropanoids and polyketides, alkaloids and derivatives, lipids and lipid-like molecules, lignans, neolignans, and related compounds. Among these, organoheterocyclic compounds constitute the largest group with 4,830 compounds. Benzene analogs are the second largest group, comprising 2,063 compounds. Conversely, organohalogen compounds and organic 1,3-dipolar compounds are the least represented, with only 12 and 48 compounds, respectively.

### Determination of data augmentation thresholds

Data augmentation can effectively enhance the model’s performance, but there is also a risk of “over-augmentation” that may lead to a decline in model performance [18–31]. The objective of this study is to determine the optimal thresholds for data augmentation to minimize the risk of “over-augmentation” as much as possible. For each data category, the dataset was divided by us into training, validation, and test sets. In the context of both SE and Topo-AL for data augmentation, a comprehensive evaluation and optimization of parameters regarding the multiples of enumeration and the similarity thresholds for augmentation have been carried out. These thresholds are determined based on the model’s performance metrics on the validation set, such as mean absolute error (MAE) and coefficient of determination (*R*^2^). As the data augmentation procedure unfolds, these performance indicators on the validation set are continuously and meticulously monitored and assessed. Once these metrics approach the predefined target limits, which indicates a likely onset of overfitting, the data augmentation process will be promptly ceased. In the liquid chromatography–mass spectrometry analysis employing a C18 chromatographic column, the C18 column exhibits a pronounced incapacity to discriminate among the majority of compounds with optical isomerism. The stereochemical information, particularly optical rotation, of the vast preponderance of compounds exerts negligible influence on the RT. Under such circumstances, the utilization of SMILES expressions for data augmentation and prediction emerges as a judicious choice, as it refrains from introducing any substantial errors or inaccuracies pertaining to stereochemistry.

#### Determination of the SE threshold

The strategic enumeration of SMILES strings [18] during the model’s predictive phase has been identified as a crucial strategy to improve model performance and reduce biases resulting from uneven data distributions. SE generates multiple noncanonical SMILES by varying the order in which atoms in the molecular graph are traversed when representing molecules with SMILES strings. Moreover, during the process of SE, new data were not generated without limitation. In the event that the error on the validation set began to rise, which signified a potential occurrence of overfitting, the parameters or strategies of data augmentation would be promptly adjusted [21]. The experimental results are presented as follows.

During the experimental phase, a comparative examination was carried out to evaluate the influence of a spectrum of multiples of SMILES string enumeration, varying from 0 to 20 times, on the model. Notably, at a 1× enumeration multiple, the model’s *R*^2^ score improved from 74.27% to 87.85%. At a 5× multiple, accuracy peaked at 99.99%, with the MAE plummeting from 67.3314 to 0.2516. Beyond the 5× threshold, the *R*^2^ score stabilized at 99.99% with negligible variance. Further enumeration up to 20 times resulted in a minor MAE reduction to 0.1590. These findings indicate that a 5× enumeration markedly bolstered model performance, yielding a 34.63% improvement. For detailed results, refer to Table S1.

The augmentation of SMILES string enumeration effectively mitigates data sparsity in model training by broadening sample diversity. This strategy broadens the model’s understanding of compound features, leading to enhanced precision in property predictions. However, the advantages of enumeration may diminish with increasing numbers of data points, potentially due to the introduction of excessive redundancy that could detract from training efficiency.

#### Determination of the Topo-AL threshold

A Topo-AL strategy has been implemented, with molecular topological fingerprints being utilized to enhance the model’s predictive accuracy regarding compound properties. For Topo-AL, compounds within a specific range of chemical structural similarity were only enumerated to prevent the generation of new data points that deviated excessively from the original data distribution. In each iteration, the impact of newly added data points on the model’s generalization ability was meticulously evaluated, and it was ensured, through cross-validation and other methods, that the model would not overfit these new data. Adding compounds with a structural similarity higher than 0.75 significantly increased the training set size, improving the model’s *R*^2^ from 0.7427 to 0.8453, while the MAE decreased from 67.3314 to 34.3671. When compounds with a similarity threshold of 0.5 were included, the dataset size doubled, and the model’s *R*^2^ further improved to 0.9831 with an MAE of 9.57. However, when introducing compounds with a lower similarity level between 0.5 and 0.1, the *R*^2^ decreased, fluctuating between 0.83 and 0.87, possibly due to the introduction of unnecessary data that interfered with the model. This suggests that a similarity threshold higher than 0.5 is optimal for enhancing generalization. The performance declines with highly dissimilar compounds may be attributed to the introduction of noisy data, which can hinder the model’s generalization ability. These findings guide the selection of the Topo-AL threshold to balance model performance and generalization ability. For detailed results, please refer to Table S2.

The combination of these 2 enhancement methods highlights the significant performance improvement achievable through a prudent augmentation strategy. Specifically, a 5-fold increase in enumeration based on SMILES strings broadens the diversity of the data, thereby enhancing the model’s predictive accuracy. Additionally, similarity enhancement targeting compounds with a 0.5 threshold or higher contributes to the model’s generalization ability. However, it is crucial to carefully calibrate the augmentation process to avoid introducing redundant or noisy information that might undermine the model’s stability and precision and cause “over-augmentation” interference. Meticulously determining the optimal enhancement strategies and parameters is essential for achieving a balance between improved performance and enhanced generalization.

### Ablation experiments on data augmentation

Ablation experiments were conducted to evaluate the impact of data augmentation on the performance of quantitative structure–retention relationship (QSRR) models. The aim was to understand the mechanisms by which different data enhancement techniques influence model performance. The investigation centered on 2 strategies: SE and Topo-AL, which incorporates similarity-based molecular topology augmentation. To ensure a fair comparison, all models were configured with consistent parameters for SE. The experiments utilized *R*^2^ to measure the models’ explanatory power regarding sample variability and MAE to assess the accuracy of predictions. These metrics collectively reflect the models’ predictive performance and fit to the data.

Ablation experiments have demonstrated that the standalone use of the SE strategy markedly improves the model’s *R*^2^ to 0.9999, reducing the MAE to 0.2516. Similarly, the application of the Topo-AL strategy alone elevates the *R*^2^ to 0.9831, with the MAE decreasing to 9.4791. However, validation against experimental data exposed notable discrepancies, suggesting overfitting with single-strategy implementations. The combined application of SE and AL strategies optimizes the model’s *R*^2^ to 0.9999 and minimizes the MAE to 0.1001, significantly enhancing the alignment with experimental outcomes. A comprehensive analysis of these results is detailed in Table S3.

**Fig. 3.**
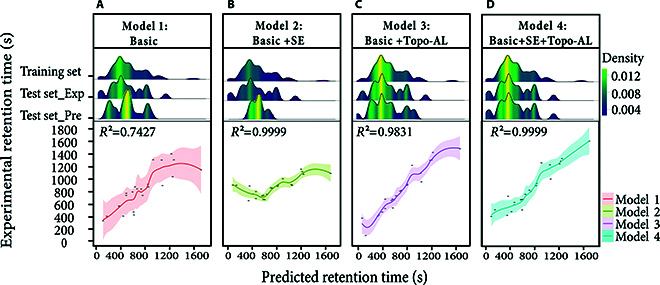
Comparative analysis of predictive performance in data ablation studies. (A) Baseline unaugmented model. (B) Model with SE to enhance data diversity. (C) Model with Topo-AL to emphasize molecular similarity. (D) Combined SE and Topo-AL strategies for potential synergistic effects.

The correlation between the test data’s predicted values and their experimental counterparts is shown in Fig. 3, which illustrates the distinct impact of various ablation strategies through 4 panels. Figure 3A shows the baseline unaugmented model. Figure 3B depicts the model with SE, enhancing data diversity. Figure 3C presents the model with Topo-AL, emphasizing molecular similarity. Figure 3D combines both strategies for potential synergistic effects. Each panel’s upper figure displays the density of RT distributions, while the lower 2D scatter plot shows the concordance between predictions and experiments, with prediction error indicated by polygons. Figure 3A’s predictions exhibit over-representation in intermediate times and inaccuracies at the extremes. Figure 3C’s Topo-AL implementation narrows the error margin, markedly enhancing correspondence. Figure 3D’s combination of SE and Topo-AL strategies results in a highly consistent distribution, indicating superior predictive performance.

The ablation study reveals that while individual enhancement methods improve performance, there are inherent limitations to their effectiveness. The combined application of SE and Topo-AL strategies achieves a synergistic effect, significantly enhancing predictive performance. This combination not only improves accuracy and reliability but also broadens and deepens the model’s learning, leading to enhanced overall performance. The study emphasizes the importance of both SE and Topo-AL strategies in QSRR modeling, where their synergistic application maximizes predictive capability.

### Real-data validation of discriminant analysis utilizing molecular fingerprints

In order to validate the practical applicability of the model, additional experiments were performed. A new set of compounds, which had been completely absent during the training process, was collected to simulate the new compounds that might be encountered in real-world applications. The dataset was composed of 100 compounds with a variety of chemical structures and properties. Upon testing with this new dataset, the total prediction error of these models was 49.52 s, and the average error was 1.59 s, showing good generalization ability and indicating that this method did not pose difficulties in predicting new compounds due to data augmentation. Among the sampled compounds, the SVR algorithm exhibited the best performance in 95% of the OPSRT models, whereas the Random Forest Regressor algorithm was effective in the remaining 5%. For a comprehensive overview, including the PubChem ID, classification model number, RT_EXP, RT_PRE, prediction error, and OPSRT parameters (such as the error range, selected algorithm, feature descriptor count, enumeration coefficient, and active learning threshold), please refer to Table S4.

**Fig. 4.**
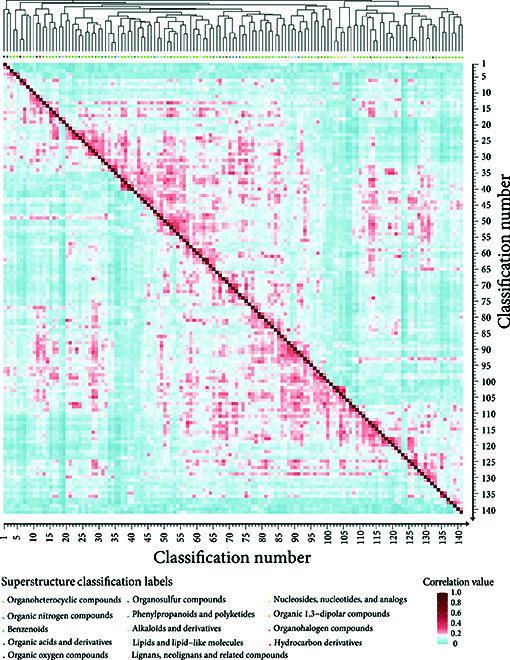
Heatmap of correlation analysis among various compound classes in RT prediction.

A visual display of the interrelationships among 141 compound categories is presented in Fig. 4, while the names and numbers of the classifications are detailed in Table S5. The heatmap in the middle part is used to depict the similarity among different categories, with its value range set between 0 and 1.0. The order of these categories is determined based on the results of the clustering algorithm employed above. Such an arrangement stands in sharp contrast to the secondary classification denoted by the color coding beneath the clustering diagram, plainly manifesting a notably significant disparity between the classification outcomes yielded by the clustering algorithm and the classification approach based on bioactive groups.

Overall, these compound categories show a relatively low similarity level. In terms of the similarity distribution, it appears rather sparse. Specifically, the first quartile (Q1) is 0.09, the median (Q2) is 0.3, and the third quartile (Q3) is 0.17. More notably, the vast majority (99.27%) of the similarity values are within the range below 0.5, and as high as 98.5% of the similarity values are concentrated in the even narrower range from 0 to 0.3, which undoubtedly fully indicates that there are extremely significant structural differences among most categories. Although these compound categories are sorted according to the clustering results, remarkably, this sorting method scatters the category members that originally belonged to the same secondary classification in our classification method. This phenomenon profoundly discloses a considerably substantial difference between the classification approach predicated on chemical structure and that founded on bioactive groups. This discovery further accentuates the disparities between the aforesaid 2 classification methods and also vehemently underlines the scientific value and practical importance of the classification methodology adopted in this research.

### Model performance evaluation and comparative analysis with existing approaches

This study conducted a comprehensive evaluation of the MTSCAM model’s performance in predicting chromatographic RTs using the extensive and publicly accessible METLIN-SMRT dataset. The evaluation aimed to compare the MTSCAM model’s effectiveness with other cutting-edge models. The assessment employed standard metrics, including MAE, mean absolute percentage error (MAPE), and coefficient of determination (*R*^2^), to measure the accuracy of the model’s predictions.

The existing RT prediction models include GNN-based models (“RT-Transformer” [32], “DeepGCN-RT” [6], and “GCN” [8]) and some advanced versions of ensemble learning methods (“DNNpwa” [22] and “GNN-RT” [9]). These models are well known for their advanced predictive capabilities in RT prediction, among which DeepGCN-RT performs the best. The performance of the MTSCAM model was also further compared with several classical ML techniques [8] that have a good track record in quantitative structure–activity relationship (QSAR) modeling.

A comparative analysis of the MTSCAM model against other methods is presented in Table. The DeepGCN-RT model, which utilizes deep graph convolutional networks and molecular graph data, is regarded as the current state of the art, with an *R*^2^ of 0.89 and an MAE of 26.55 s. However, the MTSCAM model outperforms all the benchmark models, attaining an MAE of 23.31 s and an *R*^2^ of 0.98. Compared with the state-of-the-art model, it shows a 12.20% reduction in MAE and a 10.11% improvement in *R*^2^.

**Table.** Summary of comparative performance analysis: MTSCAM model versus established models

Model	MAE (s)	MedAE (s)	MAPE	*R* ^2^	Source year
MTSCAM	23.31	8.90	0.02	0.98	This study
RT-Transformer	27.3	12.46	-	0.88	2024 [32]
DeepGCN-RT	26.55	12.38	0.03	0.89	2023 [6]
GCN	29.4	15.02	0.04	0.89	2021 [8]
DNNpwa	39.62	25.08	0.05	0.85	2021 [22]
GNN-RT	39.87	25.24	0.05	0.85	2021 [9]
1D CNN	34.7	18.7	0.04	-	2022 [34]
MLP	40.8	-	0.05	0.84	2021 [8]
RF	56.4	-	0.07	0.78	2021 [8]
SVM	45.6	-	0.06	0.82	2021 [8]
AB	58.2	-	0.07	0.76	2021 [8]
GB	111.6	-	0.15	0.40	2021 [8]

**Fig. 5.**
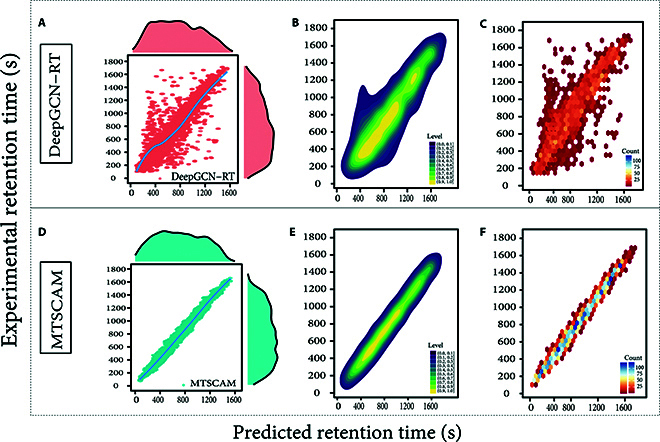
Comparative analysis of RT predictions on the SMRT dataset. (A) DeepGCN-RT model’s predictive capability shown through a statistical distribution of experimental versus predicted RTs. (B) Bivariate kernel density estimation (KDE) plots to visualize the data’s distribution characteristics. (C) Hexbin plots comparing predicted and experimental RTs, highlighting predictive consistency and potential discrepancies. (D) MTSCAM model’s performance on the same dataset, using statistical distribution of RTs. (E) KDE plots for the MTSCAM model, showing improved data distribution characteristics. (F) Hexbin plots for the MTSCAM model, demonstrating superior predictive performance and reduced outliers.

A detailed comparative analysis between the MTSCAM model and the current leading DeepGCN-RT model on the standardized SMRT dataset is shown in Fig. 5. Figure 5A illustrates the DeepGCN-RT model’s predictive capability through a statistical distribution of experimental versus predicted RTs, highlighting the model’s accuracy. Figure 5B utilizes bivariate kernel density estimation (KDE) plots to visualize the data’s distribution characteristics. Figure 5C employs hexbin plots to compare predicted and experimental RTs, graphically representing the predictive consistency and potential discrepancies of the models. Figure 5D to F replicates this analysis for the MTSCAM model across 3 distinct data categories, providing a visual comparison of performance on the same dataset. In all scatter plots, the *x* axis denotes experimental RTs, and the *y* axis denotes model-predicted times. The scatter plots in Fig. 5A and D show the correlation between predicted and actual values for the SMRT test set, with data points’ proximity to the diagonal line indicating predictive accuracy. A comparison of Fig. 5C and F reveals that the MTSCAM model’s predictions are more likely to align with the diagonal, suggesting lower prediction error and greater stability. Furthermore, the comparison between Fig. 5B and E indicates that the MTSCAM model reduces the incidence of outliers and surpasses the DeepGCN-RT model in prediction accuracy and reliability.

The MTSCAM model integrates a suite of molecular topology-based classification and data enhancement strategies, effectively advancing beyond classical models to enhance predictive performance and robustness. This study’s findings validate the effectiveness of these optimization strategies, which are designed to leverage molecular topology for the MTSCAM model. They augment the model’s capability to discern and weigh input data features and to select pertinent information while filtering out noise. These enhancements are expected to significantly bolster the model’s predictive accuracy and reliability.

### Model interpretability analysis with SHAP

**Fig. 6.**
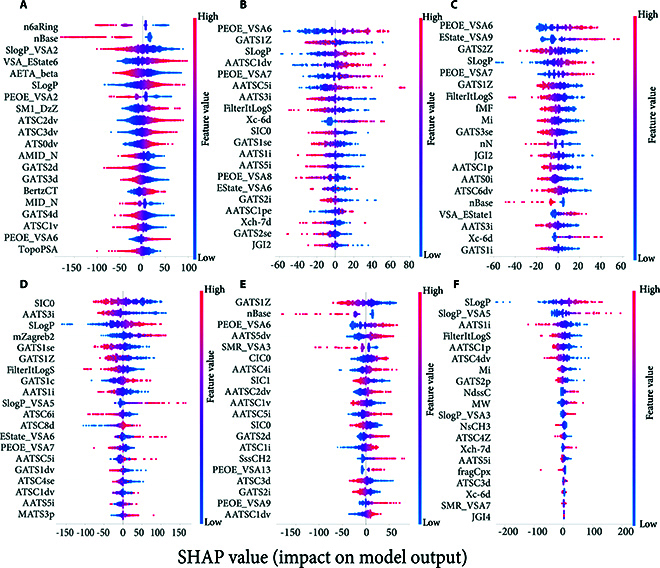
SHAP summary for the models. (A) SHAP analysis of model MTSCAM, highlighting the top 20 most influential feature descriptors, ranked by importance. (B to F) SHAP analyses of submodels B to F, focusing on feature descriptors relevant to submodels 2, 13, 44, 97, and 139, respectively.

In the scientific realm, the comprehension of the operational mechanisms of complex models is crucial. The SHAP (SHapley Additive exPlanations) is a game-theoretic approach that explains the output of any machine learning model by attributing the prediction to the contribution of each feature based on Shapley values from cooperative game theory. This method provides a fair and consistent way to evaluate the impact of each feature on the model's predictions. A comprehensive SHAP analysis of model A and submodels B to F, as illustrated in Fig. 6, is hereby presented. Model A unfolds a comprehensive vista of diverse feature dimensions, with the incorporation of features such as n6aRing, nBase, and SlogP_VSA2. This confers upon model A the capacity to apprehend the multifaceted essence of the system under scrutiny. It is conspicuous that certain features, namely, SLogP, AATS3i, GATS1Z, and PEOE_VSA6, recur across multiple models, thereby denoting their fundamental role within the model output framework. The elaborate feature assemblage of model A, while proffering a holistic perspective, concomitantly poses challenges in terms of interpretability.

Conversely, the submodels possess distinct foci. ATSC (centered Moreau–Broto autocorrelation) descriptors are a class of molecular descriptors that quantify the distribution of specific atomic properties, such as ionization potential or charge, across the molecular structure. These descriptors are calculated by centering the Moreau–Broto autocorrelation values to eliminate biases and improve the statistical reliability of the data. Model D, with its ATSC family of features (ATSC6i, ATSC8d, etc.), is oriented toward specific subprocesses or local mechanisms within the system. In model E, features like AATSC4i and SIC1 are centered around particular properties and intermolecular interactions. The SHAP value distributions offer further illumination. Model A exhibits a wide gamut (−150 to 100) of values, suggestive of the intricate interplay and dynamic equilibria among features. Features with extreme values are shown to exert a preponderant influence over predictions. Submodels B (−60 to 80) and C (−200 to 200) display disparate patterns, with the extensive range and large absolute values of model C intimating the potent nonlinear and heterogeneous impacts of its features.

The model ensemble represents a complex, multilayered structure. The diversity and overlap of features, while augmenting the informational plenitude, also precipitate an augmentation in the complexity of interpretation. The differential accents of submodels and the singularities of SHAP value distributions furnish valuable perspectives. In practical applications, model A is deployed as a comprehensive compendium for overall trends, while submodels D and E function as precise implements for dissecting specific factors. The identification of crucial features for model refinement is facilitated by a profound apprehension of SHAP values, potentially culminating in an augmentation of both predictive precision and interpretability. The subtleties in feature dimensions, model priorities, and SHAP value distributions are disclosed by the methodical exploration of the SHAP analysis of model A and submodels B to F. The foundation for model optimization, deeper mechanistic elucidation, and enhanced interpretability is thereby established by these revelations.

## Discussion

In this study, the molecular fingerprint-based MTSCAM model was constructed, which significantly improves prediction accuracy and enhances model generalizability through the integration of SE, Topo-AL strategies, and structural classification of compounds. This innovative approach enriches the model’s chemical intuition and provides a novel perspective for predicting chromatographic RTs. The average prediction error of the MTSCAM model is 23 s, which highlights the substantial impact of incorporating the structure of compounds into the training process. This is potentially more essential than the traditional focus on feature engineering, which involves selecting and modifying the features of a model.

The results of the discriminant analysis provide further validation of the model’s practical effectiveness, demonstrating the existence of variability in feature requirements across different compound classes. These findings provide valuable insights that can be used to further optimize the model. The successful application of the MTSCAM model introduces a powerful tool for chemical analysis, contributing to the advancement of chemical analysis technology. The fusion of ML with precise structural classification, coupled with innovative data enhancement techniques and sophisticated algorithms, enables highly accurate predictions of compound RTs.

The utility of RT prediction models is evident in their application to chemical analyses, including nontargeted screening, as they help reduce labor and resource requirements while improving research efficiency. Nevertheless, the conclusions drawn and the generalizability of the MTSCAM model must be validated against a wider array of chemical structures and datasets. Future research should focus on the synergistic combination and refinement of data enhancement strategies to elevate model performance and expand its applicability within cheminformatics. Moreover, enhancing the model’s interpretability and visualization will be essential, allowing chemists to more readily grasp the chemical implications of the model’s predictions and extend their utility in chemical research and industrial applications.

## Materials and Methods

### Dataset acquisition

The dataset used liquid chromatography coupled with HRMS (LC-HRMS) for RT determination. The analysis encompassed 10,905 pure standard compounds, processed in batches of 100 molecules each, differentiated by molecular weight. A Thermo Fisher Acquity Ultrahigh-performance liquid chromatography system, coupled with a Thermo ID-X quadrupole-Orbitrap high-resolution mass spectrometer, was utilized for the analysis. Chromatographic separation was achieved using an ACQUITY UPLC BEH C18 column (2.1 × 100 mm, 1.7 μm) at 45 °C with a flow rate of 0.45 ml/min. The mobile phase was a mixture of ultrapure water with 0.1% formic acid (A) and acetonitrile with 0.1% formic acid (B), following a gradient elution profile: 2% B to 99% B over 20 min, held at 99% B for 4 min, then re-equilibrated to 2% B over the next minute, and stabilized for 5 min. The Q-Orbitrap HRMS operated in positive ion mode with electrospray ionization (ESI), set at a spray voltage of 3.8 kV. The capillary and probe heater temperatures were maintained at 325 and 400 °C, respectively, with an S-lens RF (radio frequency) voltage of 60 V. Sheath, auxiliary, and sweep gas pressures were finely adjusted, and nitrogen was employed as the spray and collision gas. Full MS scans ranged from mass/charge ratio (*m*/*z*) 200 to 500 with a mass resolution of 120,000 full width at half maximum (FWHM), and the AGC (automatic gain control) target was standardized. Our analytical method has been validated for effectiveness across the majority of compounds [33]. Peak identifications were conducted manually, with RTs and additional chemical information meticulously recorded in the database.

### Data augmentation

To counteract data sparsity and class imbalance resulting from the detailed classification of compound categories, several data augmentation techniques were implemented. The goal was to augment the training dataset for underrepresented categories, enhancing the model’s predictive performance. The primary strategies utilized in this research were SE and Topo-AL, as well as their combined application.

SE involved generating additional SMILES strings for existing compounds to diversify the training data. Topo-AL, on the other hand, selectively introduced new data points based on their molecular topological similarity to existing samples, aiming to enrich the dataset with informative examples. The integration of SE and Topo-AL aimed to leverage the strengths of both methods, leading to a more robust and generalized model.

#### SMILES enumeration

The SMILES is a commonly used method for the representation of molecules in the field of cheminformatics. It is capable of producing multiple valid strings for a single compound. To address the inherent variability of SMILES strings, a comprehensive enumeration strategy was employed. This systematic approach generates diverse SMILES variants to expand the dataset and enhance sample coverage. The SE project on GitHub and the RDKit library were utilized to ensure that the chemical graph representations and the generated SMILES strings correspond accurately to the original molecules. A thorough analysis was conducted to determine the influence of various enumeration indices on model performance. Controlled and ablation experiments were performed on a targeted dataset to rigorously assess the impact of the SE strategy. The model’s predictive efficacy was evaluated using the coefficient of determination (*R*^2^) and the MAE, providing quantitative insights into the performance.

#### Topology-guided active learning

Facing the challenge of limited experimental data, an active learning strategy was embraced to bolster our dataset and, crucially, to refine the model’s generalization. The literature’s emphasis on the sensitivity of predictive error to similarity thresholds prompted the enrichment of the training dataset with structurally analogous compounds. This approach aimed to heighten the accuracy of a broader spectrum of predictions.

Topo-AL was incorporated for data augmentation to amplify the model’s grasp of molecular structural diversity. RDKit was utilized to calculate the structural Tanimoto similarity coefficients between the training set molecules and a comprehensive dataset. Compounds exceeding a 0.5 similarity threshold were selectively appended to the training set. Ablation studies substantiated the active learning strategy’s efficacy.

Extended-connectivity fingerprints (ECFPs) were selected as the molecular descriptors, with cosine similarity being employed as the preferred metric for comparison. A brute-force algorithm systematically assessed the similarity between each training set molecule and the dataset at large, enlisting molecules with a 50% or higher similarity for inclusion in the training dataset. Advanced deep learning architectures, including Siamese networks and molecular pair neural networks, were harnessed to assimilate molecular fingerprint embeddings, which were then channeled into similarity searches.

Active learning was utilized to further refine the augmented training set through SE, thereby preparing it for the subsequent model training phase. The Tanimoto similarity coefficient, pivotal for our selection process, is calculated as shown in Eq. 1:

$$\text{Tanimoto}\;s\text{imilarity}=\frac{2\times \left|A\cap B\right|}{\left|A\right|+\left|B\right|-\left|A\cap B\right|}$$ (1)

Here, *A* and *B* denote the fingerprints of 2 molecules. The dataset was successfully expanded by enumerating SMILES strings and integrating active learning based on structural similarity thresholds. This foundational work is pivotal for enhancing the model’s generalization and predictive precision, with duplicates in the test dataset initially excluded from consideration.

### Modeling methodology

In chemoinformatics, modeling the relationship between molecular structure and RT is complex due to various influencing factors, such as molecular physicochemical properties and chromatographic conditions. This study employed 7 ML algorithms for modeling the structure–RT relationship, with a focus on automated parameter optimization to enhance performance. The choice of algorithm was informed by the specific problem, data characteristics, and performance objectives. For each dataset class post-data enhancement, the algorithm with the lowest error rate was selected as the expert model for that class. Detailed training procedures are provided in the Supplementary Materials.

## Data Availability

The METLIN’s SMRT dataset can be accessed at https://doi.org/10.6084/m9.figshare.8038913. The MassFoCUS dataset can be accessed at http://scms.cau.edu.cn.
