# Supplementary material for: Development of an Efficient and Generalized MTSCAM Model to Predict Liquid Chromatography Retention Times of Organic Compounds
Source: Research (Wash D C). 2025 Feb 7;8:0607. doi: 10.34133/research.0607 (PMC11803058; doi:10.34133/research.0607)
Supplement: Supplementary 1 — Text S1 Tables S1 to S5 [file research.0607.f1.docx]

Title

Development of an Efficient and Generalized MTSCAM Model to Predict Liquid Chromatography Retention Times of Organic Compounds

**Authors**

Mengdie Fan^1^, Chenhui Sang^2, 3^, Hua Li^4^, Yue Wei^4^, Bing Zhang^4^, Yang Xing^2, 3^, Jing Zhang^2, 3^, Jie Yin^2, 3^, Wei An^4*^, and Bing Shao^1, 2, 3*^

Content of Supplementary Materials:

Text S1. Additional methodological details: classification techniques, modeling strategies, molecular topology similarity assessment, application of machine learning algorithms, model performance evaluation

Table S1. Determination of the optimal SMILES enumeration threshold: impact on model performance metrics.

Table S2. Calibration of the similarity enhancement threshold: effects on predictive accuracy.

Table S3. Ablation study results: quantitative assessment of model performance following the application of data enhancement strategies.

Table S4. Comprehensive analysis of retention times for real compounds: test results and predictive evaluation.

Table S5. Names and numbers of classification.

**Text S1. Additional methodological details: classification techniques,** **modeling strategies of OPSRT, molecular topology similarity assessment,** **model performance evaluation**

**1. Classification Techniques**

Utilizing the ClassyFire tool, the compounds in the training set were classified based on structural principles [3], [27]. The process entailed the following steps:

**SMARTS structural calculation:** ClassyFire translated input SMILES strings into SMARTS structures for feature matching within the training set.

**Parsing of SMARTS structures:** SMARTS structures were parsed into substructures or functional groups, including rings, functional groups, and bonding patterns.

**Matching of structural formulas with classification labels:** Parsed substructures were matched against a predefined set of classification labels, following a hierarchical system and rules established by researchers.

**Determination of matching categories and weights:** Matched substructures were assigned categories and weights based on the classification system and predefined weighting rules, often linked to the importance of non-hydrogen atoms.

**Integration of classification and weights:** The categories and weights were integrated to form the overall classification for each compound, typically using a weighted summation method.

**Final classification outcome:** The classification process involves identifying the parent class based on the weights assigned. The feature with the highest weight is designated as the parent class. In cases where the dominant feature has a weight of two or greater, a detailed consideration is given to the total count of structural elements, including rings, heterocycles, ring atoms, heteroatoms within the rings, halogen atoms, fused rings, and other heteroatoms, as indicated by the node structure keys. This comprehensive assessment underscores the significance of each feature within the classification framework. The culmination of this integrated approach allows for the determination of the compound's final classification, typically prioritizing the category with the preeminent weight. The methodology is encapsulated by equation (S1):

$f\left( X_{i} \right)=arg\max_{C}\left( \sum_{j=1}^{N} w_{j}\cdot I\left( C_{j},X_{i} \right) \right)$ (S1)

In this equation, $f\left( X_{i} \right)$ represents the final classification result of the compound, $X_{i}$ represents the SMARTS structure of the compound, $C_{j}$ represents the $j_{th}$ category, $w_{j}$ represents the weight of the $j_{th}$ category, $I\left( \cdot\right)$ is the indicator function, which returns 1 if the match is successful, and 0 otherwise. This equation encapsulates the process where each substructure is matched against all possible classification labels, followed by a weighted summation of these matches. The result is a quantitative score for each category, and the category with the highest score is determined to be the final classification of the compound.

## 2. Modeling strategies of OPSRT

For each augmented classification dataset, we employed the Mordred package within RDKit, extracting features from SMILES strings that encode properties like molecular size, shape, polarity, and charge distribution. From an initial set of 1600 potential features, we applied a stringent filtration process, removing those present in over 90% of compounds, missing in 75% of samples, or with a Pearson Correlation Coefficient (PCC) above 0.9, to reduce multicollinearity. This refined the feature set to 300 to 400 descriptors. Feature importance was further assessed using a 500-tree random forest model, facilitating the recursive feature elimination with cross-validation to enhance model predictive power. The machine learning pipeline, integrated within RDKit, was tailored for robust molecular descriptor analysis, leveraging a suite of algorithms selected for their analytical efficacy. The algorithms used are as follows:

**Bayesian Ridge Regression (RFF)**, an advanced regression method that seamlessly merges the probabilistic framework of Bayesian inference with the regularization technique of ridge regression. The essence of the model is encapsulated in the succinct formulation of equation (S2):

$Y=X\beta+\epsilon$ (S2)

In this formulation, $Y$ denotes the dependent variable, the focal point of our predictive endeavors. The matrix $X$ comprises the independent variables, the array of characteristics that sculpt the landscape of our analysis. The vector $\beta$ represents the regression coefficients, the pivotal parameters that quantify the relationship between variables. The term $\epsilon$ symbolizes the error, an embodiment of the stochastic variability inherent in our observations, which is posited to adhere to a normal distribution with a mean of 0 and variance $\sigma^{2}: \epsilon\sim N\left( 0, \sigma^{2} \right)$.

Bayesian Ridge Regression endows the regression coefficients with a prior distribution, typically a Gaussian distribution, as expressed in $\beta\sim N\left( 0,\lambda^{-1}I \right)$, with $I$ being the identity matrix and $\lambda$ serving as the regularization parameter within ridge regression. This prior distribution is a critical component that reflects our initial beliefs about the parameters before observing the data.

Employing Bayes' theorem, a fundamental tenet of Bayesian inference, we derive the posterior distribution, a reflection of our updated understanding of the parameters in light of the observed data. This is elegantly represented in equation (S3):

$p\left( \beta|Y,X \right)\propto p\left( Y|X,\beta\right)\times p\left( \beta\right)$ (S3)

Here, $p\left( Y|X,\beta\right)$ is the likelihood function, encapsulating the probability of observing the data $Y$ for a given set of parameters $\beta$, which is conventionally assumed to be normally distributed.

**LASSO Regression**, known formally as the Least Absolute Shrinkage and Selection Operator, is a linear regression model renowned for its ability to perform both variable selection and regularization. This dual functionality is accomplished by minimizing a loss function that includes L1 regularization, which is pivotal in conferring the model its ability to shrink coefficients and select significant predictors. The core principle of LASSO is elegantly expressed in the following equation (S4):

$L\left( \alpha,\lambda\right)=\sum_{i=1}^{n} \left( y_{i}-X_{i}\alpha\right)^{2}+\lambda\parallel\alpha\parallel_{1}$ (S4)

In this equation, $y_{i}$ represents the true value of the dependent variable for the ith observation. $X_{i}$ denotes the feature vector associated with this observation, a constellation of independent variables. The vector $\alpha$ comprises the regression coefficients, the parameters that the model seeks to estimate. The parameter $\lambda$ is the regularization term, a hyperparameter that modulates the trade-off between the model's fit to the data and its complexity. The $L1$ norm, represented by $\parallel\alpha\parallel_{1}$, is the key to LASSO's ability to perform both variable selection and regularization, as it penalizes the absolute values of the coefficients, thereby driving some to zero and effectively selecting a simpler model.

**Random Forest (RF)**, an ensemble learning technique that amplifies model performance by combining multiple decision trees. This approach leverages the aggregate predictions of a forest of 500 trees, each grown from a random subset of the data. The robustness of the ensemble is derived from the diversity of these individual trees, with the final prediction being a consensus reached through their combined output. The process of building each decision tree is succinctly described by the following equation (S5):

$T_{b}="\text{BuildTree"}\left( D_{b},"\text{feature\_subset"} \right)$ (S5)

Here, $T_{b}$ represents the construction of the $b$th decision tree using a bootstrap sample $D_{b}$and a subset of features referred to as $“\text{feature\_subset”}$. The predictive function of the ensemble is elegantly expressed in equation (S6):

$f\left( x \right)=\frac{1}{B}\sum_{b=1}^{B} T_{b}\left( x \right)$ (S6)

Here, $F\left( x \right)$ represents the averaged prediction, the consensus reached by all trees $T_{b}\left( x \right)$ in the forest.

**Adaptive Boosting (AB)**, an ensemble technique that incrementally builds a sequence of decision trees, each iteration of which refines the model by focusing on the instances misclassified in the previous round. This method adaptively adjusts the weights of training samples, particularly increasing the weights of those that are more challenging to classify correctly. This process is designed to improve the model's performance on the hardest-to-classify cases. The key step in each iteration, where the weight of the $t$th tree is determined, is expressed by the following equation (S7):

$\alpha_{t}=\frac{1}{2}\ln\left( \frac{1-\epsilon_{t}}{\epsilon_{t}} \right)$ (S7)

Here, $\alpha_{t}$ signifies the weight accorded to the $t$th iteration, a measure that reflects the model's confidence in the predictions of the current tree. The term $\epsilon_{t}$ denotes the error rate of the iteration, calculated as the proportion of samples that have been misclassified. The logarithmic transformation ensures that the weight $\alpha_{t}$ is directly proportional to the accuracy of the tree, with more accurate trees receiving larger weights.

**Gradient Boosting (GB)**, an ensemble technique that enhances model performance through the sequential integration of decision trees, each designed to address the residual errors of its predecessors. This method iteratively refines the model by adjusting the weights of the samples that were misclassified in the previous iteration, thereby concentrating the model's efforts on the more challenging cases. The incremental learning process is encapsulated in equation (S8):

$F_{t}\left( x \right)=F_{t-1}\left( x \right)+\alpha_{t}h_{t}\left( x \right)$ (S8)

In this equation, $F_{t}\left( x \right)$ represents the updated prediction function after the $t$th tree has been incorporated. $h_{t}\left( x \right)$ is the prediction of the new tree added in the $t$th iteration, and $\alpha_{t}$ is the weight assigned to this tree, which is determined by the gradient descent optimization process. This weight reflects the contribution of the new tree to the overall model and is adjusted to minimize the loss function.

**Support Vector Regression (SVR)**, an extension of the support vector machine framework, is adept at predicting continuous values by determining the optimal decision boundary for data segregation within a regression scenario. The model's fundamental formulation is encapsulated in the following equation (S9):

$f\left( x \right)=\left\langle w,x \right\rangle+b$ (S9)

Here, $f\left( x \right)$ denotes the predicted value, $\left\langle w,x \right\rangle$ represents the dot product of the weight vector $w$ and the input vector $x$, capturing the linear relationship between features. The term $b$ is the bias, adjusting the prediction to align with the data's distribution.

The SVR model adeptly addresses nonlinear relationships within the data by employing a Radial Basis Function (RBF) kernel, a kernel function renowned for its versatility and efficacy in capturing the subtle complexities of data. The performance of the SVR model is critically dependent on two key hyperparameters: $C$ and $\gamma$. The hyperparameter $C$ manages the trade-off between the model's smoothness and the number of support vectors, thereby controlling the regularization strength of the model. Conversely, $\gamma$ dictates the influence of individual samples on the RBF kernel, effectively setting the breadth of the kernel's effect.

In the quest for optimal model performance, the SVR model meticulously navigates the hyperparameter space, considering a wide range of values for $C$ and $\gamma$, typically spanning from 0.001 to 1000. Hyperparameter tuning is conducted through the use of Grid Search, a comprehensive search algorithm that systematically explores the predefined parameter grid to identify the combination of hyperparameters that yields the highest model efficacy. This thorough optimization process ensures that the SVR model is precisely attuned to the data's characteristics, enhancing both its predictive accuracy and robustness.

**Artificial Neural Network (ANN)**, is a sophisticated computational model inspired by the complexity of the human brain, capable of recognizing and interpreting complex patterns through extensive data-driven learning. Comprising a network of interconnected neurons, each neuron processes input signals into meaningful outputs through a combination of weighted connections and activation functions. The fundamental computation performed by a neuron to produce its output $y$ is captured by the following equation (S10):

$y=\sigma\left( \sum_{i} w_{i}x_{i}+b \right)$ (S10)

In this equation, $w_{i}$ represents the weight assigned to the $i$th input, a coefficient that modulates the influence of each signal. $x_{i}$ denotes the input signal itself, the datum that propagates through the network. $b$ is the bias, an adjustable parameter that provides flexibility in the neuron's response. $\sigma$ symbolizes the activation function, a mathematical transformation that introduces non-linearity into the model, enabling it to capture intricate relationships within the data. The activation function $\sigma$ is pivotal, often employing functions such as the sigmoid, hyperbolic tangent, or rectified linear unit (ReLU), each serving to regulate the flow of information and to introduce the complex decision boundaries that allow the ANN to excel in pattern recognition and prediction tasks.

To objectively assess the efficacy of each model, a 5-fold cross-validation paradigm was meticulously implemented. This approach entails the division of the dataset into five distinct subsets, each serving a turn as the validation cohort while the complementing segments amalgamate to form the training repertoire. Within the grid search purview, this methodology is reiterated for every constellation of hyperparameters, affording the model an opportunity to undergo training and subsequent validation. The model's prowess is quantified by the absolute temporal error and the R² metric, derived from the model's predictions on the validation fold.

The ensemble of steps, from data augmentation to feature selection and machine learning orchestration, was encapsulated within a scikit-learn pipeline, with the average performance metrics, such as the mean square error (MSE) and the coefficient of determination (R²), being calculated for each. The algorithm that demonstrated the most exceptional performance on the validation set was anointed as the ultimate model. This selection process was facilitated by the automated tool, scikit-learn's GridSearchCV, which systematically traversed the hyperparameter space to identify the optimal combination. The OPSRT for each class of compounds was trained utilizing the aforementioned algorithms, ensuring a rigorous and systematic approach to model development.

**3. Molecular topology similarity assessment**

This study employed a gated network to select the optimal prediction sub-models (OPSRTs) that are best suited for each predictive task. The core function of the gated network is to evaluate the performance of multiple OPSRTs during the prediction process and to choose the most appropriate model for new molecular inputs based on these assessments.

Initially, categorical information was encoded into numerical one-hot encodings, where a new column was created for each category, assigned a value of 1 for membership and 0 otherwise. Subsequently, we prepared a dictionary containing SMILES strings and corresponding weight vectors. For each compound in the dictionary, a corresponding one-dimensional weight vector was generated based on its classification outcome, representing the weights of each OPSRT. The SMILES strings were converted into Extended Connectivity Fingerprints (ECFP) as input for the model. The dimensionality of these molecular fingerprint feature vectors $X$ was $D$ as shown in equation (S11).

$X\in\mathbb{R}^{D}$ (S11)

A neural network model was designed with molecular fingerprints as input and a weight vector equal in number to the OPSRTs as output, as shown in equation (S12). The hidden layer $H$ of the model was computed through a weight matrix $W$, bias vector $b$, and activation function $\sigma$. The updating mechanism for the weights is delineated by equation (S13), while the biases are adjusted in accordance with equation (S14), where $\alpha$ is the learning rate, and $\nabla_{W}L$ and $\nabla_{b}L$ are the gradients of the loss function $L$ with respect to the weights $W$ and bias $b$, respectively. The gated network facilitated the calculation of the initial probability distribution $G$, and the final layer used a Softmax activation function to map the gated network's output $G$ to the number of OPSRTs, ensuring that the output was a valid probability distribution $P$, as shown in Equations (S15). The model was trained using labeled training set data. The model's predictions were measured against actual labels using a cross-entropy loss function, and the model's weights were updated using a gradient descent algorithm. The update mechanism for weights and biases ensured that the model could dynamically select the most appropriate OPSRT based on the input molecular fingerprint feature vector $X$.

$G=\sigma\left( g\left( H \right) \right)$ (S12)

$W_{\text{new}}=W_{\text{old}}-\alpha\cdot\nabla_{W}L$ (S13)

$b_{\text{new}}=b_{\text{old}}-\alpha\cdot\nabla_{b}L$ (S14)

$P=\text{Softmax}\left( VG+c \right)$ (S15)

The model's performance was evaluated on the validation and test sets using accuracy, precision, recall, and F1 scores. Through the weight allocation mechanism of the gated network, the model was able to dynamically select the optimal OPSRT for each predictive task, thereby enhancing the accuracy and efficiency of the predictions.

**4. Model performance evaluation**

In this study, a suite of model evaluation metrics was employed to quantitatively assess the accuracy of machine learning model predictions. These metrics included the coefficient of determination ($R^{2}$), mean absolute error (MAE), median absolute error (MedAE), and mean absolute percentage error (MAPE).

**Coefficient of Determination (**$\boldsymbol{R}^{\boldsymbol{2}}$**):** The $R^{2}$ metric quantifies the degree to which the predicted values align with the actual observations. It ranges from 0 to 1, with values closer to 1 indicating superior model performance. The calculation is detailed in equation (S16):

$R^{2}=1-\frac{\sum_{i=1}^{n} \left( y_{i}-\hat{y}_{i} \right)^{2}}{\sum_{i=1}^{n} \left( y_{i}-y \right)^{2}}$ (S16)

Here $y_{i}$ represents the $i_{th}$ actual observation, $\hat{y}_{i}$ is the $i_{th}$ predicted value, and $y$ is the mean of the observed values.

**Mean Absolute Error (MAE):** The MAE represents the average magnitude of the errors in a set of predictions, without considering their direction. It is calculated as the mean of the absolute differences between prediction and actual values, as shown in equation (S17):

$MAE=\frac{1}{n}\sum_{i=1}^{n} \left| y_{i}-\hat{y}_{i} \right|$ (S17)

where $n$ is the total number of observations.

**Median Absolute Error (MedAE):** The MedAE is the median of the absolute prediction errors, offering a more robust measure against outliers compared to the MAE. It is expressed in equation (S18):

$MedAE=\text{Median}\left( \left| y_{i}-\hat{y}_{i} \right| \right)$ (S18)

Here Median indicates the median value of the absolute errors.

**Mean Absolute Percentage Error (MAPE):** The MAPE is a measure of prediction accuracy that expresses the magnitude of the error in percentage terms relative to the actual values. It is calculated using equation (S19):

$MAPE=\frac{100\%}{n}\sum_{i=1}^{n} \left| \frac{y_{i}-\hat{y}_{i}}{y_{i}} \right|$ (S19)

where n is the number of observations. Note that MAPE is not applicable for all cases where the actual value is zero, since the denominator cannot be zero. It is important to note that MAPE is undefined for cases where the actual value $y_{i}$ is zero, as the denominator must not be zero.

Each of these metrics provides a unique perspective on model performance, and their combined use allows for a comprehensive evaluation of the model's predictive capabilities. Additionally, validation against real-world data was conducted to ensure the robustness of the model's performance assessment.

# Table S1. Determination of the optimal SMILES enumeration threshold: impact on model performance metrics.

| SE-I | R^2^ | MAE |
| --- | --- | --- |
| 0 | 0.7427 | 67.3314 |
| 1 | 0.8785 | 64.3711 |
| 2 | 0.9750 | 7.7886 |
| 3 | 0.9907 | 2.9488 |
| 4 | 0.9948 | 19.4107 |
| **5** | **0.9999** | **0.2516** |
| 8 | 0.9999 | 0.2516 |
| 10 | 0.9999 | 0.2516 |
| 20 | 0.9999 | 0.1590 |

SE-I, SMILES enumeration index.

# Table S2. Calibration of the similarity enhancement threshold: effects on predictive accuracy.

| AL-I | R^2^ | MAE |
| --- | --- | --- |
| 0 | 0.7427 | 67.3314 |
| 0.75 | 0.8453 | 34.3671 |
| **0.5** | **0.9831** | **9.5791** |
| 0.4 | 0.8523 | 17.1475 |
| 0.25 | 0.8527 | 20.3722 |
| 0.1 | 0.8451 | 24.3714 |

AL-I, active learning index.

# Table S3. Ablation study results: quantitative assessment of model performance following the application of data enhancement strategies.

|  | choice | | | | $R^{2}$ | $\mathrm{MAE}$ |
| --- | --- | --- | --- | --- | --- | --- |
| Model1: Basic | ✓ |  |  |  | 0.7427 | 63.3470 |
| Model2: Basic+ SE |  | ✓ |  |  | 0.9999 | 0.2516 |
| Model3: Basic+ AL |  |  | ✓ |  | 0.9831 | 9.4791 |
| Model4: Basic+ SE+ AL |  |  |  | ✓ | 0.9999 | 0.1001 |

SE, SMILES enumeration. AL-I, active learning.

# Table S4. Comprehensive analysis of retention times for real compounds: test results and predictive evaluation.

| ID | Model number | Error_min(s) | Error_max(s) | Algorithm | Number of features | SE-I | AL-I | RT_True | RT_Pre | Error |
| --- | --- | --- | --- | --- | --- | --- | --- | --- | --- | --- |
| 76 | 16 | 0.89 | 9.56 | RandomForest Regressor | 17 | 5 | 0.5 | 1118.7 | 1110.14 | -8.5577 |
| 173 | 2 | 0.01 | 14.34 | SVR | 64 | 5 | 0.5 | 770.4 | 757.586 | -12.814 |
| 183 | 2 | 0.01 | 14.34 | SVR | 64 | 5 | 0.5 | 971.9 | 973.954 | 2.05383 |
| 269 | 9 | 0.23 | 45.7 | SVR | 163 | 5 | 0.5 | 766.4 | 732.092 | -34.308 |
| 283 | 13 | 0.61 | 37.39 | SVR | 62 | 5 | 0.5 | 1200.2 | 1177.03 | -23.174 |
| 313 | 2 | 0.01 | 14.34 | SVR | 64 | 5 | 0.5 | 746.8 | 754.87 | 8.06986 |
| 546 | 9 | 0.23 | 45.7 | SVR | 163 | 5 | 0.5 | 731.9 | 775.121 | 43.2209 |
| 688 | 2 | 0.01 | 14.34 | SVR | 64 | 5 | 0.5 | 978 | 981.637 | 3.63738 |
| 777 | 12 | 0.28 | 24.86 | SVR | 42 | 4 | 0.5 | 629.9 | 643.669 | 13.7691 |
| 873 | 1 | 0.09 | 8.9 | SVR | 19 | 4 | 0.5 | 777.7 | 771.787 | -5.9128 |
| 988 | 3 | 0.15 | 36.66 | RandomForest Regressor | 233 | 6 | 0.5 | 362.00 | 394.422 | 32.4218 |
| 1001 | 9 | 0.23 | 45.7 | SVR | 163 | 5 | 0.5 | 493.50 | 454.688 | -38.812 |
| 1016 | 2 | 0.01 | 14.34 | SVR | 64 | 5 | 0.5 | 1101.5 | 1089.93 | -11.57 |
| 1126 | 2 | 0.01 | 14.34 | SVR | 64 | 5 | 0.5 | 1228.5 | 1225.16 | -3.3432 |
| 1175 | 14 | 0.12 | 15.37 | SVR | 12 | 5 | 0.5 | 672.1 | 658.236 | -13.864 |
| 1181 | 13 | 0.61 | 37.39 | SVR | 62 | 5 | 0.5 | 626.4 | 641.288 | 14.8885 |
| 1197 | 2 | 0.01 | 14.34 | SVR | 64 | 5 | 0.5 | 865 | 874.327 | 9.32744 |
| 1280 | 7 | 0.06 | 34.71 | SVR | 19 | 4 | 0.5 | 720.3 | 687.82 | -32.48 |
| 1354 | 2 | 0.01 | 14.34 | SVR | 64 | 5 | 0.5 | 722.3 | 731.371 | 9.07076 |
| 1386 | 22 | 0.78 | 45.56 | SVR | 92 | 6 | 0.5 | 1132.7 | 1133.26 | 0.56204 |
| 1532 | 26 | 0.02 | 39.09 | SVR | 95 | 5 | 0.5 | 703.7 | 686.095 | -17.605 |
| 1566 | 25 | 0.07 | 46.97 | SVR | 44 | 4 | 0.5 | 754.6 | 753.994 | -0.6061 |
| 1880 | 9 | 0.23 | 45.7 | SVR | 163 | 5 | 0.5 | 940.3 | 947.616 | 7.31639 |
| 1965 | 2 | 0.01 | 14.34 | SVR | 64 | 5 | 0.5 | 694.6 | 706.994 | 12.3938 |
| 2056 | 8 | 0.87 | 29.47 | SVR | 36 | 4 | 0.5 | 1102.4 | 1089.78 | -12.62 |
| 2095 | 2 | 0.01 | 14.34 | SVR | 64 | 5 | 0.5 | 1093.7 | 1100.67 | 6.97037 |
| 2188 | 22 | 0.78 | 45.56 | SVR | 92 | 6 | 0.5 | 929.4 | 961.328 | 31.9284 |
| 2218 | 23 | 0.61 | 18.77 | SVR | 30 | 4 | 0.5 | 670.2 | 679.988 | 9.78799 |
| 2271 | 23 | 0.61 | 18.77 | SVR | 30 | 4 | 0.5 | 902.7 | 914.235 | 11.5347 |
| 2285 | 15 | 0.65 | 15.32 | SVR | 37 | 5 | 0.5 | 871 | 860.966 | -10.034 |
| 2298 | 2 | 0.01 | 14.34 | SVR | 64 | 5 | 0.5 | 953.7 | 955.576 | 1.87571 |
| 2491 | 12 | 0.28 | 24.86 | SVR | 42 | 4 | 0.5 | 601.6 | 581.242 | -20.358 |
| 2587 | 3 | 0.15 | 36.66 | RandomForest Regressor | 233 | 6 | 0.5 | 369.70 | 381.447 | 11.747 |
| 2614 | 15 | 0.65 | 15.32 | SVR | 37 | 5 | 0.5 | 739 | 730.854 | -8.1464 |
| 2623 | 20 | 0.22 | 91.38 | RandomForest Regressor | 100 | 5 | 0.5 | 578.6 | 570.016 | -8.5841 |
| 2860 | 9 | 0.23 | 45.7 | SVR | 163 | 5 | 0.5 | 576.9 | 578.951 | 2.05108 |
| 2881 | 6 | 0.15 | 15.07 | SVR | 102 | 6 | 0.5 | 936.8 | 939.934 | 3.13396 |
| 3269 | 4 | 0.1 | 8.23 | SVR | 35 | 4 | 0.5 | 196.80 | 204.996 | 8.1964 |
| 3271 | 25 | 0.07 | 46.97 | SVR | 44 | 4 | 0.5 | 1175.3 | 1132.45 | -42.846 |
| 3419 | 24 | 0.96 | 79.74 | SVR | 49 | 4 | 0.5 | 687.8 | 726.797 | 38.997 |
| 3535 | 23 | 0.61 | 18.77 | SVR | 30 | 4 | 0.5 | 887.1 | 877.862 | -9.2385 |
| 3609 | 17 | 0.21 | 36.36 | SVR | 44 | 5 | 0.5 | 638.4 | 621.788 | -16.612 |
| 3896 | 14 | 0.12 | 15.37 | SVR | 12 | 5 | 0.5 | 789.7 | 795.552 | 5.85178 |
| 3945 | 6 | 0.15 | 15.07 | SVR | 102 | 6 | 0.5 | 584 | 592.439 | 8.4389 |
| 3952 | 8 | 0.87 | 29.47 | SVR | 36 | 4 | 0.5 | 945.2 | 953.886 | 8.68576 |
| 4024 | 26 | 0.02 | 39.09 | SVR | 95 | 5 | 0.5 | 510.9 | 534.692 | 23.792 |
| 4112 | 10 | 0.49 | 17.03 | SVR | 37 | 4 | 0.5 | 591.4 | 594.301 | 2.90078 |
| 4148 | 8 | 0.87 | 29.47 | SVR | 36 | 4 | 0.5 | 597.6 | 599.287 | 1.68652 |
| 4177 | 12 | 0.28 | 24.86 | SVR | 42 | 4 | 0.5 | 917.2 | 924.652 | 7.45241 |
| 4476 | 11 | 0.08 | 23.58 | SVR | 51 | 4 | 0.5 | 877.8 | 873.507 | -4.293 |
| 4478 | 22 | 0.78 | 45.56 | SVR | 92 | 6 | 0.5 | 882.2 | 906.734 | 24.5341 |
| 4758 | 7 | 0.06 | 34.71 | SVR | 19 | 4 | 0.5 | 932.2 | 936.538 | 4.33751 |
| 4808 | 21 | 0.1 | 54.24 | SVR | 29 | 4 | 0.5 | 901.3 | 950.826 | 49.5256 |
| 4846 | 2 | 0.01 | 14.34 | SVR | 64 | 5 | 0.5 | 1121.4 | 1118.25 | -3.1484 |
| 4889 | 7 | 0.06 | 34.71 | SVR | 19 | 4 | 0.5 | 666.5 | 681.28 | 14.7803 |
| 5089 | 11 | 0.08 | 23.58 | SVR | 51 | 4 | 0.5 | 573.3 | 576.144 | 2.84376 |
| 5238 | 12 | 0.28 | 24.86 | SVR | 42 | 4 | 0.5 | 681.2 | 681.946 | 0.74556 |
| 5681 | 18 | 0.31 | 16.28 | SVR | 35 | 4 | 0.5 | 770.4 | 772.54 | 2.14033 |
| 6054 | 2 | 0.01 | 14.34 | SVR | 64 | 5 | 0.5 | 677.1 | 679.197 | 2.09674 |
| 6092 | 2 | 0.01 | 14.34 | SVR | 64 | 5 | 0.5 | 1036.8 | 1050.94 | 14.144 |
| 6225 | 2 | 0.01 | 14.34 | SVR | 64 | 5 | 0.5 | 720.9 | 707.946 | -12.954 |
| 6296 | 13 | 0.61 | 37.39 | SVR | 62 | 5 | 0.5 | 667.8 | 697.381 | 29.581 |
| 6370 | 26 | 0.02 | 39.09 | SVR | 95 | 5 | 0.5 | 617.1 | 632.349 | 15.2485 |
| 6576 | 12 | 0.28 | 24.86 | SVR | 42 | 4 | 0.5 | 1104 | 1117.65 | 13.6545 |
| 6714 | 22 | 0.78 | 45.56 | SVR | 92 | 6 | 0.5 | 579.9 | 579.873 | -0.0269 |
| 6733 | 2 | 0.01 | 14.34 | SVR | 64 | 5 | 0.5 | 885.5 | 888.312 | 2.81181 |
| 6751 | 12 | 0.28 | 24.86 | SVR | 42 | 4 | 0.5 | 822.6 | 844.053 | 21.4533 |
| 6887 | 2 | 0.01 | 14.34 | SVR | 64 | 5 | 0.5 | 967.7 | 962.911 | -4.7892 |
| 7145 | 9 | 0.23 | 45.7 | SVR | 163 | 5 | 0.5 | 887.5 | 909.628 | 22.1277 |
| 7273 | 7 | 0.06 | 34.71 | SVR | 19 | 4 | 0.5 | 594.1 | 593.863 | -0.2371 |
| 7277 | 18 | 0.31 | 16.28 | SVR | 35 | 4 | 0.5 | 607.1 | 603.873 | -3.2271 |
| 7411 | 23 | 0.61 | 18.77 | SVR | 30 | 4 | 0.5 | 658.9 | 645.336 | -13.564 |
| 7465 | 2 | 0.01 | 14.34 | SVR | 64 | 5 | 0.5 | 805.3 | 811.299 | 5.99867 |
| 7478 | 2 | 0.01 | 14.34 | SVR | 64 | 5 | 0.5 | 917.2 | 917.069 | -0.131 |
| 7523 | 7 | 0.06 | 34.71 | SVR | 19 | 4 | 0.5 | 842.5 | 833.145 | -9.355 |
| 7528 | 4 | 0.1 | 8.23 | SVR | 35 | 4 | 0.5 | 731.4 | 735.891 | 4.49105 |
| 7692 | 2 | 0.01 | 14.34 | SVR | 64 | 5 | 0.5 | 1211.2 | 1199.02 | -12.178 |
| 7699 | 6 | 0.15 | 15.07 | SVR | 102 | 6 | 0.5 | 659 | 666.111 | 7.11051 |
| 7832 | 18 | 0.31 | 16.28 | SVR | 35 | 4 | 0.5 | 743.9 | 739.687 | -4.2134 |
| 7891 | 2 | 0.01 | 14.34 | SVR | 64 | 5 | 0.5 | 740.3 | 728.719 | -11.581 |
| 8005 | 2 | 0.01 | 14.34 | SVR | 64 | 5 | 0.5 | 969 | 980.126 | 11.1262 |
| 8033 | 1 | 0.09 | 8.9 | SVR | 19 | 4 | 0.5 | 564.7 | 558.663 | -6.0369 |
| 8121 | 26 | 0.02 | 39.09 | SVR | 95 | 5 | 0.5 | 620.9 | 646.905 | 26.005 |
| 8175 | 11 | 0.08 | 23.58 | SVR | 51 | 4 | 0.5 | 722 | 714.047 | -7.9528 |
| 8265 | 18 | 0.31 | 16.28 | SVR | 35 | 4 | 0.5 | 666.6 | 673.413 | 6.81332 |
| 8299 | 12 | 0.28 | 24.86 | SVR | 42 | 4 | 0.5 | 1082 | 1091.61 | 9.60849 |
| 8333 | 3 | 0.15 | 36.66 | RandomForest Regressor | 233 | 6 | 0.5 | 442.40 | 443.938 | 1.53849 |
| 8575 | 8 | 0.01 | 14.34 | SVR | 64 | 5 | 0.5 | 1026.2 | 1017.19 | -9.0088 |
| 8606 | 22 | 0.78 | 45.56 | SVR | 92 | 6 | 0.5 | 885.7 | 864.939 | -20.761 |
| 8723 | 17 | 0.21 | 36.36 | SVR | 44 | 5 | 0.5 | 236.50 | 249.722 | 13.2225 |
| 8792 | 2 | 0.01 | 14.34 | SVR | 64 | 5 | 0.5 | 1178.5 | 1167.56 | -10.939 |
| 8931 | 18 | 0.31 | 16.28 | SVR | 35 | 4 | 0.5 | 802 | 807.122 | 5.12194 |
| 8982 | 5 | 0.15 | 15.07 | SVR | 102 | 6 | 0.5 | 659.4 | 661.672 | 2.2721 |
| 9600 | 18 | 0.31 | 16.28 | SVR | 35 | 4 | 0.5 | 549.6 | 536.209 | -13.391 |
| 9732 | 11 | 0.08 | 23.58 | SVR | 51 | 4 | 0.5 | 755.8 | 735.409 | -20.391 |
| 9952 | 9 | 0.23 | 45.7 | SVR | 163 | 5 | 0.5 | 857.4 | 852.243 | -5.1567 |
| 10322 | 12 | 0.28 | 24.86 | SVR | 42 | 4 | 0.5 | 822.4 | 828.581 | 6.18064 |
| 10378 | 14 | 0.12 | 15.37 | SVR | 12 | 5 | 0.5 | 892.2 | 898.344 | 6.1445 |
| 10639 | 19 | 0.9 | 6.79 | SVR | 40 | 4 | 0.5 | 901.6 | 907.81 | 6.21032 |
| 10710 | 5 | 0.15 | 15.07 | SVR | 102 | 6 | 0.5 | 1021.2 | 1012.18 | -9.0198 |

SE-I, SMILES enumeration index. AL-I, active learning index. RT_True, experiment retention time. RT_Pre, prediction retention time

# Table S5. Names and numbers of classification.

| Classification number | Claddification name | Classification number | Claddification name | Classification number | Claddification name |
| --- | --- | --- | --- | --- | --- |
| 1 | Organic carbonic acids and derivatives | 51 | Diazinanes | 101 | Pyrans |
| 2 | Carboxylic acids and derivatives | 52 | Naphthalenes | 102 | Purine nucleosides |
| 3 | Epoxides | 53 | Benzoxazoles | 103 | Pyrrolopyrimidine nucleosides and nucleotides |
| 4 | Imidazopyrimidines | 54 | Coumarans | 104 | Pyrimidine nucleosides |
| 5 | Triazines | 55 | Benzothiepins | 105 | 5'−deoxyribonucleosides |
| 6 | Organooxygen compounds | 56 | Benzoxazepines | 106 | Prenol lipids |
| 7 | Allyl−type 1,3−dipolar organic compounds | 57 | Tetrahydroisoquinolines | 107 | Steroids and steroid derivatives |
| 8 | Pyrrolopyrimidines | 58 | Strychnos alkaloids | 108 | Triazolodiazepines |
| 9 | Pyrrolopyrazines | 59 | Pyrrolopyridines | 109 | Diazepanes |
| 10 | Lactams | 60 | Azoles | 110 | Thiadiazines |
| 11 | Thioethers | 61 | Fatty Acyls | 111 | Azolidines |
| 12 | Aryl halides | 62 | Isoquinolines and derivatives | 112 | Furans |
| 13 | Phenol ethers | 63 | Benzopyrazoles | 113 | Organonitrogen compounds |
| 14 | Heteroaromatic compounds | 64 | Oxazinanes | 114 | Triazolopyridines |
| 15 | Benzisoxazoles | 65 | Phenylpropanoic acids | 115 | Triazolopyridazines |
| 16 | Triazolopyrimidines | 66 | Benzimidazoles | 116 | Azaspirodecane derivatives |
| 17 | Benzo−1,2,3−triazines | 67 | Cinnamic acids and derivatives | 117 | Tropane alkaloids |
| 18 | Flavonoids | 68 | Benzene and substituted derivatives | 118 | Pyridopyrimidines |
| 19 | Dibenzylbutane lignans | 69 | Tropones | 119 | Pyrroles |
| 20 | Pyrrolo[3,4−d]pyridazines | 70 | Isoflavonoids | 120 | Thienopyrroles |
| 21 | Pteridines and derivatives | 71 | Linear 1,3−diarylpropanoids | 121 | Pyridines and derivatives |
| 22 | Benzoxazolines | 72 | Diarylheptanoids | 122 | Imidazodiazepines |
| 23 | Pyranodioxins | 73 | Homoisoflavonoids | 123 | Lupin alkaloids |
| 24 | Stilbenes | 74 | Pyridopyrazines | 124 | Organic sulfonic acids and derivatives |
| 25 | Pyrrolines | 75 | Pyridodiazepines | 125 | Pyrazolopyrimidines |
| 26 | Benzopyrans | 76 | Benzotriazoles | 126 | Diazines |
| 27 | Naphthopyrans | 77 | Quinolines and derivatives | 127 | Pyrrolidines |
| 28 | Anthracenes | 78 | Benzoxadiazoles | 128 | Harmala alkaloids |
| 29 | Indenes and isoindenes | 79 | Benzothiazoles | 129 | Azetidines |
| 30 | Benzothiophenes | 80 | Benzothiadiazoles | 130 | Benzothiazines |
| 31 | Phenol esters | 81 | Isoxazolopyridines | 131 | Benzodiazepines |
| 32 | Neoflavonoids | 82 | Pyrrolotriazines | 132 | Peptidomimetics |
| 33 | Diazanaphthalenes | 83 | Furo[2,3−d]pyrimidines | 133 | Quinolizidines |
| 34 | Thienopyrimidines | 84 | Protoberberine alkaloids and derivatives | 134 | Azepines |
| 35 | 2−arylbenzofuran flavonoids | 85 | Naphthofurans | 135 | Azepanes |
| 36 | Ergoline and derivatives | 86 | Indoles and derivatives | 136 | Oxanes |
| 37 | Benzofurans | 87 | Fluorenes | 137 | Piperidines |
| 38 | Benzothiazepines | 88 | Aryltetralin lignans | 138 | Thiophenes |
| 39 | Benzoxazines | 89 | Amaryllidaceae alkaloids | 139 | Triazolopyrazines |
| 40 | Naphthothiazoles | 90 | Cephalotaxus alkaloids | 140 | Imidolactams |
| 41 | Coumarins and derivatives | 91 | Aurone flavonoids | 141 | Imidazothiazoles |
| 42 | Isoindoles and derivatives | 92 | Imidazo[1,2−a]pyrazin−8−ones |  |  |
| 43 | Thienopyridines | 93 | Benzodioxoles |  |  |
| 44 | Piperazinopiperidines | 94 | Benzodioxanes |  |  |
| 45 | Thienopyridazines | 95 | Imidazopyridines |  |  |
| 46 | Quinolidines | 96 | Morphinans |  |  |
| 47 | Indanes | 97 | Phenols |  |  |
| 48 | Ibogan−type alkaloids | 98 | Tetralins |  |  |
| 49 | Eburnan−type alkaloids | 99 | Cycloheptathiophenes |  |  |
| 50 | Benzazepines | 100 | Pyrazolopyridines |  |  |
